# Learning through connections: clinical participation and interpersonal relationships in longitudinal integrated clerkships and traditional block rotations in Taiwan

**DOI:** 10.1186/s12909-024-05120-y

**Published:** 2024-02-10

**Authors:** Po-Kai Chan, Yung-Chih Wang, Shih-Chung Huang, Yaw-Wen Chang

**Affiliations:** 1 Department of Internal Medicine, Tri-Service General Hospital, National Defense Medical Center, Taipei, Taiwan; 2 Division of Infectious Diseases and Tropical Medicine, Department of Internal Medicine, Tri- Service General Hospital, National Defense Medical Center, Taipei, Taiwan; 3 Department of Internal Medicine, Kaohsiung Armed Forces General Hospital, Kaohsiung, Taiwan; 4 Department of Family and Community Medicine, Tri-Service General Hospital, National Defense Medical Center, No.325, Sec. 2, Cheng-Gong Rd., Neihu Dist, Taipei City, 11490 Taiwan

**Keywords:** Longitudinal integrated clerkship, Ecomap, Workplace learning, Situated learning, Interpersonal relationship, Clinical participation

## Abstract

**Background:**

Longitudinal integrated clerkships (LICs) and traditional block rotations (TBRs) employ different designs that provide various learning experiences for students. In this study, we explored students’ clinical participation and interpersonal interactions in LICs and TBRs at 2 metropolitan hospitals in Taiwan.

**Methods:**

In April 2018, we enrolled 15 LIC and 29 TBR students. We conducted a cross-sectional survey which required the students to outline a typical daily schedule during their internal medicine rotations and draw an ecomap of the clinical team members. With the patient in the center as a reference, the size of each circle in an ecomap indicated the importance of the member; the distances and number of connecting lines between two circles represented the relationship and frequency of interaction, respectively, between the corresponding members. We analyzed the results and compared the responses of the LIC and TBR students.

**Results:**

The LIC students spent more time on direct patient care and in the outpatient clinic/operation room, whereas the TBR students participated more in educational activities and in observation behind their seniors. In the ecomap analysis, the LIC students had a closer relationship with attending physicians and had better interactions with patients and preceptors than did the TBR students. Conversely, the TBR students felt closer to and interacted more frequently with interns and residents.

**Conclusions:**

The LIC students had more opportunities to care for patients directly and engaged in interactions with patients and attending physicians more frequently than did the TBR students.

**Trial registration:**

Ethical approval for the study was obtained from the Institutional Review Board of Tri-Service General Hospital (TSGHIRB 2–106–05–018).

**Supplementary Information:**

The online version contains supplementary material available at 10.1186/s12909-024-05120-y.

## Background

Longitudinal integrated clerkships (LICs), curricula emphasizing continuity and fostering relationships among medical students, patients, and physicians for a transformative learning experience, have been widely adopted in many countries and contexts [1, 2]. LICs provide students with continuity of supervision and patient care and enable simultaneous learning across different disciplines through integrated curriculum design [1, 3]. Given these features and the additional benefits of longitudinal placement, the LIC model provides a unique learning experience that ensures students develop clinical competence and relationships with clinical team members [4].

The workplace learning theory and situated learning theory, associated with concepts of communities of practice and legitimate peripheral participation, have been applied to studies of learning in clinical environments, especially in LIC communities [5–10]. The workplace learning theory focuses on workplace affordances and learner agency. A clinical workplace that provides adequate opportunities, guidance, and tools has a great impact on students’ active participation in patient care [11]. Previous studies exploring the clinical activity participation and teamwork experience found that students in LIC spend more time on meaningful direct patient care activities [7], play more active doctor-like roles, and feel more integrated into the clinical team [6, 12, 13]. Longitudinal clinical placement enables students to develop skills by participating in meaningful clinical activities [1].

Longitudinal placement also provides an opportunity for students to build an interpersonal rapport with patients and clinical team members. By engaging and building rapport with team members and patients, medical students could break the so-called epistemic boundaries and enact their membership of the community, which could eventually help students’ journey from the periphery into full participation [8, 14]. LICs help establish an intimate rapport with the clinical team members, making students familiar with the settings and norms of clinical practice [15] and shaping their moral identities as future doctors [16]. A multicenter quality study also revealed that students who developed longitudinal relationships with their patients developed an increased sense of responsibility toward their care [17]. In a recent narrative review, O’Doherty et al. synthesized the data of multiple studies and concluded that the LIC model facilitates the development of meaningful relationships among students, clinical teachers, and patients, which is the main reason for successful learning within the context of an LIC [4].

Despite previous findings, most LICs are conducted in rural areas or urban community settings in Western countries [2]. Only a few medical schools have offered LICs in urban tertiary teaching hospitals, yet most have employed outpatient clinics as the primary training settings [12, 18–22]. In Taiwan, most medical students undergo TBRs in inpatient wards at metropolitan tertiary teaching hospitals; however, Taiwan’s National Defense Medical Center (NDMC) has introduced the LIC model at the Tri-Service General Hospital (TS-LIC)—a tertiary-hospital-based inpatient-predominant LIC located in the Taipei metropolitan area [23, 24]—since 2010. While most studies comparing the clinical participation and interpersonal relationships of LIC and TBR students have been conducted in Western countries and various program contexts, from rural clinics and community health centers to urban tertiary hospitals [4, 6, 7, 12, 13], it highlights the need to understand how curriculum models affect students’ experiences within the East Asian context to complete our understanding of these topics in the LIC community.

On the other hand, there were limitations in the data-collecting methods of the existing literature. Previous studies comparing LICs and TBRs, including ours, have used interviews or participant observation to explore students’ participation in clinical activities and the experiences of students working in clinical teams, approaches that require considerable time and the guidance of trained researchers [6, 7, 12, 13, 22]. Other studies have reviewed logbooks and have calculated the number of activities or patient encounters with student involvement. However, this method has limitations related to low response rates, variation among the tasks or patient encounters recorded, and poor student engagement due to long study periods [5, 13]. To overcome the limitations of the methodology, we need a more intuitive, instant, and effective way for data collection.

To address these research gaps and based on our previous findings, we compared the clinical participation, interpersonal relationships, and interaction patterns of LIC and TBR students in tertiary hospital settings by using a self-developed questionnaire consisting of a daily schedule survey and an ecomap.

## Methods

### Curricula, sites, and settings

**Fig. 1:**
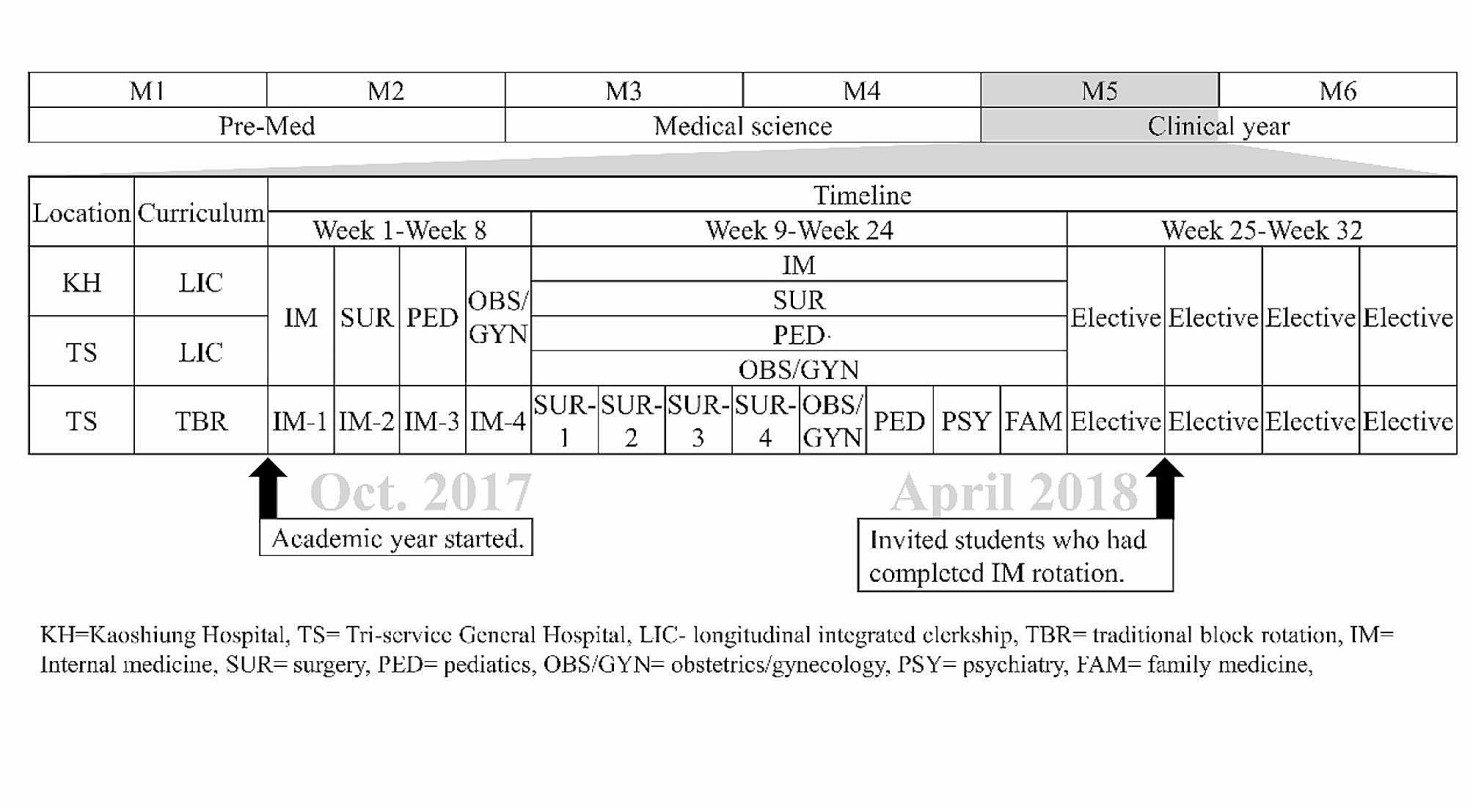
Curriculum design and data collection timeline

NDMC offers both TBRs and blended-type LICs as clinical curricula during the penultimate year of their 6-year medical school program (Fig. 1). LIC design requires an integrated clerkship to cover the learning objectives of multiple disciplines simultaneously, while blended LICs include all or most disciplines, using complementary rotations to complete the academic year. The LICs at NDMC include an immersion stage of a 2-week rotation in four major disciplines (surgery, internal medicine, obstetrics/gynecology, and pediatrics), followed by a 4-month integrated stage where students participate in the comprehensive care of patients over time and have continuing learning relationships with these patients’ clinician in each discipline longitudinally. In our model, each student is paired with preceptors from different disciplines by the program director. On the other hand, TBR students rotate in different disciplines in 2-week intervals, encountering various attending physicians or clinical teams that are randomly assigned. The key distinction lies in the structure of the LIC, where students have a more sustained and integrated experience across multiple disciplines compared to the sequential and randomly assigned rotations in the TBR model.

In the 2017/18 academic year, in addition to the Tri-Service General Hospital in Taipei (TS-LIC), Kaohsiung Armed Forces General Hospital in Kaohsiung (southern Taiwan) was selected as the second LIC site (KH-LIC). Both hospitals are tertiary teaching hospitals and situated in metropolitan areas. Fifteen medical students voluntarily applied for LICs this year, twelve and three of whom were assigned to TS-LIC and KH-LIC, respectively. All the TBR students completed their TBRs at Tri-Service General Hospital.

We selected internal medicine (IM) as the area of study. IM education plays a unique and crucial role in helping medical students understand clinical reasoning, the care of adult patients with complex conditions, and interprofessional collaboration, which are competencies that are essential in multiple specialties [25]. Previous studies investigating clinical participation have mostly focused on surgical or mixed settings; few have explored clinical participation in IM settings alone [7, 13]. Because several researchers have discussed the transformation of undergraduate IM curricula [25, 26], focusing on LICs and TBRs in IM settings enabled us to gain greater insights into students’ experiences with learning and facilitated students to build a rapport with clinical team members.

The settings of IM rotations were different between the three programs. TS-LIC students completed their IM placements in a hospitalist-run ward, where the routine care team consisted of hospitalists, nursing practitioners, and registered nurses. A few residents would briefly rotate in this ward. KH-LIC students had their IM placements in the general medical ward. The care team consisted of physicians, residents, and registered nurses, but no interns. The TBR student remained rotating in 2-week intervals in four IM specialist medical wards. The care team consisted of physicians, residents, nursing practitioners, registered nurses, and interns.

We recruited five hospitalists at TS-LIC and four IM physicians at KH-LIC to serve as IM preceptors and, as mentioned, paired each student with at least one preceptor. To ensure that the preceptors understood the objectives and practices of the LIC program, we conducted at least two sessions of faculty training at each hospital before the academic year started. One session was a one-hour overview of the curriculum, and another was a one-hour consensus meeting. The TBR students would encounter 5 to 6 clinical teachers during their IM rotations.

### Participants and research schedule

Approximately 10% of the class, or around 12 students, voluntarily join the LICs program through a lottery selection. The participants were fifth-year medical students during the 2017/18 academic year. All fifteen LIC students, including both TS-LIC and KH-LIC students, were enrolled in this study. To recruit TBR students, we adopted convenience sampling and invited 29 students during the research schedule in April 2018. We excluded students who had not completed any IM rotations.

### Measurements

We used an anonymous two-part questionnaire to collect data.

In the first part, we constructed a table with ten common clinical activities:

*Morning Meeting, Ward Round, Direct Patient Care, Senior Shadowing, Clinical Administration, Educational Activity, Informal Discussion, Outpatient Departments or Operation Room (OPD/OR), Hand-off*, and *Self-directed Learning*. The students recalled and wrote the approximate time they spent on those activities (from 7:00 am to 5:00 pm) during IM placement. We elaborate on the content of each activity in the following paragraph.

The daily IM rotation schedule usually begins with a *Morning Meeting*, a routine meeting involving case-based discussions in the conference room. Following the meeting, students usually participate in a *Ward Round*, which is a service or teaching round led by attending physicians or residents. During the ward round, patients may report new complaints, require further investigation, or be approved for discharge; each of these situations corresponds to additional patient care activities, such as history taking, physical examination, and procedure implementation (nasogastric tube insertion, urinary catheter insertion, etc.), as well as other care activities involving direct interaction with patients. *Direct Patient Care* refers to the care activities performed by the medical students under supervision. If the students simply observe behind the residents or interns performing these care activities, we categorized their observational activities as *Senior Shadowing*. In addition to these activities, seniors may supervise the completion of *Clinical Administrative* tasks by students, such as writing notes, retrieving examination reports, and entering medical orders (prescriptions or examinations) into the hospital information system. Students may also engage in *Informal Discussions* with members of the clinical team regarding various issues related to their primary care patients. (Discussions during ward rounds and about non–primary care patients are not included in this category.) At the end of the day, students usually participate in *Hand-off* activities led by chief residents or superiors.

In addition to inpatient ward activities, students may participate in patient care in outpatient departments, examination rooms, or operation rooms, referred to hereafter as *OPD/OR* activities. (Because students do not participate in OPD/OR activities every day, we calculated the time each student spent on OPD/OR activities as the average time they spent participating in such activities each week [5 weekdays]). In addition, students may participate in *Educational Activities*, which are structured learning activities including lecturing, small-group discussions, or clinical skill laboratories. Otherwise, students may arrange their own learning schedule for the remaining time, which is referred to herein as *Self-directed Learning*.

In the second part of the survey, we adopted the ecomap method, first developed by Ann Hartman in 1978 based on ecological system theory and family tree mapping, which have been widely used in healthcare research in the past two decades [27–29]. As a visualization tool, ecomaps enable users to illustrate social contexts and networks, express themselves using methods beyond verbal language, and represent multiple dimensions of their experiences [30]. We modified the ecomap methodology proposed by Ann Hartman in this study to examine the clinical team ecosystem, or a “community of practice” in situated learning theory [9], from the student perspective.

**Fig. 2:**
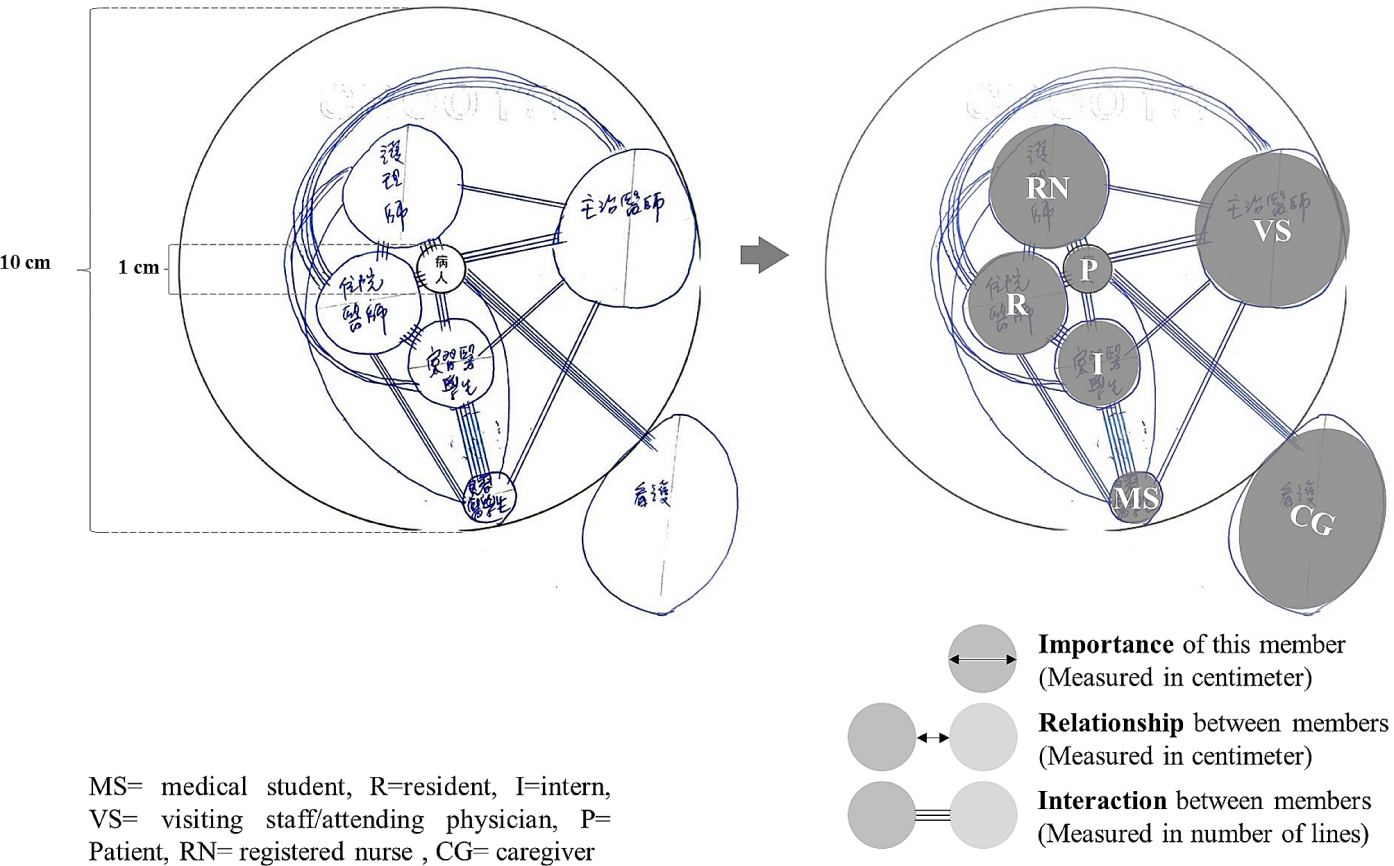
Ecomap examples and interpretations

The revised version involves the use of an ecomap featuring clinical team members (as shown in Fig. 2), with the patient positioned at the center as a point of reference. The outer circle, with a radius of ten centimeters, delineates the limits of the clinical team. The size of each circle (in centimeters) denotes the importance of the corresponding member, with a larger circle indicating greater significance. The distance between any two circles (in centimeters) reflects the relationship between the respective members, with a shorter distance indicating a closer connection. The number of connecting lines between two circles represents the frequency of interaction between the corresponding team members.

### Data collection process and eco-map measurement

We conducted this cross-sectional study in April 2018, the second-to-last month of the students’ clerkship courses. The invited students gathered in a group at a conference room, where the investigators instructed the students to fill out the survey and draw an ecomap. We explained the elements mentioned above to the participants and helped them if they encountered difficulties in filling out the survey or constructing the ecomap.

Measurement of circular sizes is typically conducted in centimeters. However, when an ellipse is encountered, the determination of its size requires a different approach. In this case, we calculate the average radius, which is defined as the mean value of its semi-major axis (a) and semi-minor axis (b). The formula used to calculate the average radius is (a + b)/2. This method provides a comprehensive measure that accounts for both the longer and shorter dimensions of the ellipse, resulting in an accurate and representative measure of its size.

### Statistical analysis

We report all the collected data as descriptive statistics (means and standard deviations). We used Fisher’s exact test to analyze categorical variables because it was previously validated for small sample sizes [31]. In addition, we conducted a Kruskal–Wallis test to compare the TS-LIC, KH-LIC, and TBR students in relation to the dependent variables of interest, namely, time spent on specific activities on a typical IM rotation day and the importance of and relationships and interactions among clinical team members. A *p*-value of ≤ 0.05 was considered statistically significant. We report eta-squared values as estimates of effect size. All the statistical analyses were performed using IBM SPSS Statistics for Windows, version 28.0.

The utilization of modified version of the ecomap methodology in this study has not undergone validation and no baseline reference is currently available for the distance or number of connecting lines between members. To address this concern, we conducted a reanalysis of the data where we adjusted the shortest distance between circles (relationships) in each ecomap into Z-scores based on the mean and standard deviation of all distances within the ecomap. Additionally, the number of the connecting lines between each pair of circles (interactions) was replaced with the ratio of that number to the total number of lines within the ecomap. This methodology aims to provide a more accurate and objective representation of the relationships and interactions among the team members.

## Results

Forty-four students (12 TS-LIC, 3 KH-LIC, and 29 TBR students) participated in this study. The average age of the participants was 23.2 ± 0.89 years, and 31 (70.5%) of the students were male; no significant differences were identified in the gender ratio among the three groups (*p* = 0.529).

### Participation in clinical activities

Our analysis of the students’ daily schedules revealed that LIC students (of both TS-LIC and KH-LIC) spent most of their time on OPD/OR activities and ward rounds, both of which are patient-oriented activities (Table 1). By contrast, the TBR students spent the most time on educational activities, self-directed learning, and senior shadowing, all of which are minimally patient-oriented.

**Table 1:** Daily schedule and ecomap data of TBR and LIC students

	Overall			Site-model									Kruskal Wallis test			Effect size	
				KH-LIC			TS-LIC			TS-TBR							
	Mean	SD	*N*	Mean	SD	*N*	Mean	SD	*N*	Mean	SD	*N*	χ	df	*p* value	Eta	Eta^2
**Participation of clinical activities (hrs)**																	
Morning meeting	1.02	0.28	44	1.00	0.00	3	0.88	0.31	12	1.09	0.27	29	4.621	2	0.099	0.331	0.109
Educational activity	1.47	0.64	43	1.17	0.29	3	1.13	0.43	12	1.64	0.68	28	6.097	2	0.047	0.384	0.148
Ward round	1.33	0.53	44	1.33	0.76	3	1.46	0.62	12	1.28	0.47	29	0.757	2	0.685	0.154	0.024
Informal discussion	0.67	0.36	39	0.83	0.58	3	0.67	0.25	12	0.64	0.39	24	0.919	2	0.632	0.139	0.019
Direct patient care	0.67	0.45	40	1.25	0.35	2	1.04	0.54	12	0.46	0.22	26	16.672	2	0.000	0.657	0.432
OPD/OR	1.30	0.99	41	1.83	0.76	3	1.80	0.98	12	1.01	0.93	26	7.816	2	0.020	0.392	0.154
Senior shadowing	1.19	0.82	44	0.67	0.29	3	0.71	0.33	12	1.44	0.89	29	9.618	2	0.008	0.432	0.187
Clinical Administration	0.63	0.32	44	0.50	0.00	3	0.54	0.26	12	0.68	0.35	29	1.002	2	0.606	0.228	0.052
Hand-off	0.44	0.36	36	0.50	0.00	2	0.30	0.42	10	0.50	0.34	24	2.701	2	0.259	0.249	0.062
Self-directed learning	1.47	0.93	43	0.83	0.58	3	1.46	0.96	12	1.54	0.95	28	1.906	2	0.386	0.192	0.037
**Importance (cm, the larger, more important)**																	
MS	1.19	0.48	44	1.17	0.45	3	1.09	0.41	12	1.23	0.51	29	0.873	2	0.646	0.127	0.016
I	2.05	0.74	29			0			0	2.05	0.74	29					
R	2.67	1.12	36	2.30	0.69	3	1.70	0.24	4	2.84	1.16	29	5.764	2	0.056	0.339	0.115
VS	3.31	1.16	44	2.80	1.35	3	2.83	0.55	12	3.56	1.28	29	3.285	2	0.193	0.300	0.090
RN	2.26	1.04	42	1.53	0.15	3	1.72	0.54	10	2.53	1.11	29	6.535	2	0.038	0.387	0.149
NP	2.19	0.84	17			0	2.03	0.64	12	2.58	1.19	5	0.717	1	0.397	0.307	0.094
**Relationship (cm, the lower, the closer)**																	
MS-P	2.35	1.92	44	1.53	1.37	3	1.30	0.98	12	2.87	2.08	29	5.316	2	0.070	0.383	0.147
MS-VS	2.81	2.12	44	1.23	1.23	3	0.85	0.71	12	3.79	1.92	29	19.788	2	0.000	0.649	0.421
MS-I	1.40	1.62	29			0			0	1.40	1.62	29					
MS-R	2.56	2.11	36	1.47	0.85	3	2.34	2.18	4	2.71	2.20	29	0.945	2	0.623	0.168	0.028
MS-NP	2.28	2.60	16			0	1.45	1.30	12	4.75	4.09	4	2.136	1	0.144	0.568	0.322
MS-RN	3.26	1.82	42	3.33	0.68	3	2.60	1.35	10	3.48	2.01	29	1.673	2	0.433	0.206	0.042
**Interaction (number of lines, the higher, more frequently)**																	
MS_P	2.21	1.42	38	2.67	1.53	3	2.83	1.47	12	1.83	1.30	23	6.122	2	0.047	0.342	0.117
MS_I	4.45	3.04	29			0			0	4.45	3.04	29					
MS_R	2.39	1.23	31	3.00	0.00	3	2.67	1.15	3	2.28	1.31	25	1.970	2	0.373	0.191	0.036
MS_VS	2.41	1.46	39	4.00	2.65	3	3.58	1.24	12	1.63	0.71	24	19.801	2	0.000	0.691	0.478
MS_RN	1.27	0.59	15	2.00		1	1.50	0.71	2	1.17	0.58	12	4.694	2	0.096	0.394	0.155
MS_NP	2.85	1.34	13			0	3.00	1.49	10	2.33	0.58	3	0.480	1	0.489	0.217	0.047

KH = Kaohsiung Armed Forces General Hospital, TS = Tri-Service General Hospital, LIC = longitudinal integrated clerkship, TBR = traditional block rotation, SD = standard deviation, N = number of students, hrs = hours, cm = centimeter, OPD/OR = outpatient clinic or operation room, P = patient, MS = medical student, R = resident, I = intern, RN = registered nurse, NP = nurse practitioner, VS = visiting staff/attending physician

The time spent on educational activities, direct patient care, OPD/OR activities, and senior shadowing differed significantly among the three groups. The LIC students spent more time on direct patient care (TS-LIC: 1.04 ± 0.54 h, KH-LIC: 1.25 ± 0.35 h) than did the TBR students (0.46 ± 0.22 h, *p* < 0.001). The LIC students also spent more time participating in OPD/OR activities (TS-LIC: 1.80 ± 0.98 h, KH-LIC: 1.83 ± 0.76 h) than did the TBR students (1.01 ± 0.93 h, *p* = 0.02). By contrast, the TBR students spent more time shadowing interns and residents (1.44 ± 0.89 h) than did the LIC students (TS-LIC: 0.71 ± 0.33 h, KH-LIC: 0.67 ± 0.29 h, *p* = 0.008). The TBR students spent the most time on educational activities (1.64 ± 0.68 h) and spent significantly more time on educational activities than did the LIC students (TS-LIC: 1.13 ± 0.43 h, KH-LIC: 1.17 ± 0.29 h, *p* = 0.047).

## Perceived importance, interpersonal relationships, and interactions

The students’ perceived importance of clinical team members was similar among the three groups; however, the perceived importance of nurses differed significantly among the groups (*p* = 0.038). All the students in both groups rated the attending physicians as the most important clinical team members, followed by residents or nursing practitioners and nurses. The average circle size for nurses in the TBR group (2.53 ± 1.11 cm) was significantly larger than those in both LIC groups (TS-LIC: 1.72 ± 0.54 cm, KH-LIC: 1.53 ± 0.15 cm). The circle sizes for attending physicians, residents, and interns did not differ significantly among the groups.

Regarding interpersonal relationships, the LIC students were the closest to the attending physicians, followed by the patients and residents/nursing practitioners. By contrast, the TBR students were the closest to the interns, followed by the patients and residents/nursing practitioners, and were not as close to the attending physicians. Comparing between groups, the average distance between the students and attending physicians in the TS-LIC and KH-LIC groups (0.85 ± 0.71 and 1.23 ± 1.23 cm, respectively) was significantly shorter than that in the TBR group (3.79 ± 1.92 cm, *p* < 0.001). Although the difference was not statistically significant, we noted that the students in both LIC groups were closer to the patients, nurses, and residents/nursing practitioners than the TBR students.

As for interactions among team members, the frequency with which the students interacted with attending physicians and patients differed significantly among the groups. The LIC students interacted most frequently interacted with attending physicians, followed by residents/nursing practitioners and patients. By contrast, the TBR students interacted the most frequently with interns (4.45 ± 3.04 lines) followed by residents/nursing practitioners and patients. Comparing between groups, the LIC students had more interaction with attending physicians (TS-LIC: 3.58 ± 1.24 lines, KH-TIC: 4.00 ± 2.65 lines, and TBR: 1.63 ± 0.71 lines, *p* < 0.001) and patients (TS-LIC: 2.83 ± 1.47 lines, KH-LIC: 2.67 ± 1.53 lines, and TBR: 1.83 ± 1.30 lines, *p* = 0.047) than did the TBR students.

The adjusted data, which shows similar results, is shown in Table 2. Compared to the TBR, the LIC students reported having more interaction with attending physicians and the patients, and closer relationships with the former.

**Table 2:** Adjusted interpersonal relationship and interaction data

	Overall			Site-model												Kruskal Wallis test			Effect size	
				KH-LIC				TS-LIC				TS-TBR								
	Mean	SD	*N*	Mean		SD	*N*	Mean		SD	*N*	Mean	SD	*N*	χ		df	*p* value	Eta	Eta^2
**Relationship (Z score†)**																				
MS-P	0.178	0.694	44	0.0389		1.1182	3	− 0.0739		0.6167	12	0.2966	0.6767	29	2.752		2	0.253	0.243	0.059
MS-VS	0.576	1.040	44	− 0.3314		0.6734	3	− 0.4476		0.3136	12	1.0937	0.8808	29	21.095		2	0.000	0.700	0.490
MS-I	-0.406	0.733	29				0				0	− 0.4060	0.7332	29						
MS-R	0.355	0.910	36	− 0.0698		0.2585	3	0.9666		1.3612	4	0.3145	0.8703	29	1.767		2	0.413	0.268	0.072
MS-NP	0.337	0.973	16				0	0.1527		0.9216	12	0.8911	1.0359	4	2.485		1	0.115	0.339	0.115
MS-RN	0.996	0.884	42	1.7006		0.2703	3	1.3957		0.7569	10	0.7853	0.8933	29	6.104		2	0.047	0.370	0.137
**Interaction (Ratio‡)**																				
MS_P	0.06	0.04	38	0.09		0.05	12	0.07		0.04	3	0.04	0.03	23	16.114		2	0.000	0.537	0.288
MS_I	0.10	0.05	29				0				0	0.10	0.05	29						
MS_R	0.05	0.03	31	0.08		0.06	3	0.08		0.02	3	0.05	0.02	25	5.219		2	0.074	0.469	0.220
MS_VS	0.07	0.06	39	0.12		0.07	12	0.09		0.03	3	0.04	0.02	24	23.280		2	0.000	0.700	0.490
MS_RN	0.03	0.01	15	0.02		0.01	2	0.04			1	0.02	0.01	12	1.715		2	0.424	0.328	0.108
MS_NP	0.07	0.03	13	0.08		0.03	10				0	0.05	0.02	3	1.849		1	0.174	0.345	0.119

KH = Kaohsiung Armed Forces General Hospital, TS = Tri-Service General Hospital, LIC = longitudinal integrated clerkship, TBR = traditional block rotation, SD = standard deviation, N = number of students, P = patient, MS = medical student, R = resident, I = intern, RN = registered nurse, NP = nurse practitioner, VS = visiting staff/attending physician

† Shortest distances between circles in an ecomap were converted into Z-scores according to the mean and standard deviation of all distances within the ecomap

‡ Number of the connecting lines between each pair circles in an ecomap was replaced by the ratio of that number to the total number of lines within the ecomap

## Discussion

In this study, we adopted a survey comprising a daily schedule outline and an ecomap to compare the clinical participation and interpersonal relationships of LIC and TBR students at metropolitan tertiary teaching hospitals. We discovered that the LIC students spent more time on patient-related activities such as ward rounds and direct patient care than the TBR students. Regarding interpersonal relationships, the LIC students had closer relationships and interacted more frequently with attending physicians. In contrast, the TBR students had closer relationships with interns but interacted less frequently with patients. The adjusted data also reassured similar results of interpersonal relationships.

Our previous studies found different experiences and perceptions between LIC and TBR students. We interviewed students in both groups and concluded that LIC students received mostly guidance and support from the attendings, whereas TBR students frequently interacted with interns [24]. LIC students also had more opportunities to build relationships and interact with their patients over time, see coherent disease and treatment progress, and eventually lead to meaningful roles in care. TBR students, in contrast, described more opportunistic learning through observation, while the short period of rotation prevented them from interacting more with the patients. This affordance provided perspective on the patients’ experience of illness and may have offered students more meaningful roles in care, which is triangulated by another quality research of our team, in which the patients perceived students providing care facilitation, companionship, and empathy [32]. In the present study, we fill the gap in how students interact with and relate to other team members and time allocation in the workplace that affords their daily learning.

Our findings regarding clinical participation are consistent with those of a previous study, in which LIC students were determined to spend a significantly higher percentage of session time working directly with patients (25%) than did TBR students in outpatient (12%) and inpatient (7%) settings [7]. In the present study, the TBR students spent considerable time on indirect patient care activities, such as observing clinical practices, delivering case presentations, or engaging in discussions, in inpatient settings. These findings support that the LIC model fosters students’ more intensive and meaningful participation in patient care [33]. The longitudinal curriculum design of the LIC model also provides LIC students with the opportunity to repeatedly engage with the same preceptors and patients, ensuring the continuity of supervision and care. In one study, LIC students were significantly more likely to experience continuity of care of patients with 34% of their patients returning to them, whereas only 5% of patients did so for TBR students [7]. The continuity of patient care helps students develop a compassionate, patient-centered professional identity [22], and the continuity of supervision enables students to earn preceptors’ trust and actively engage in patient care [6].

In addition, we discovered that the LIC students spent significantly more time in ambulatory settings than did the TBR students in the present study. A national survey of IM programs at medical schools in the United States revealed that although the clerkship directors of TBR students reported that their students were required to spend time in IM-specific ambulatory settings more often than did LIC students (44% vs. 33%), the LIC students actually spent more half days in ambulatory medicine than did the TBR students [25]. Because the LIC model promotes continuity in a students’ relationships with preceptors and patients, it promotes the educational continuity of ambulatory training and helps students engage in meaningful patient care activities [13, 34]. In another study comparing the participation of LIC and TBR students in surgical clinical activities, the researchers discovered that the LIC students recorded most of their surgical encounters (40.6%) in clinical settings, in which they participated in surgical clinical activities more actively. By contrast, the TBR students recorded most of their surgical encounters in the hospital inpatient setting (52%), wherein they mainly played observation roles [5]. Despite the NDMC LIC model being an inpatient–predominant model that differs from the ambulatory–predominant LIC models often adopted in Western countries, our model still provides LIC students with more ambulatory training opportunities and engages students in clinical practice.

In this study, we noted that, unlike LIC students, TBR students spent the most time on senior shadowing. Moreover, they interacted the most frequently and had the closest relationships with interns. Previous studies have reported that junior doctors frequently took the responsibility of teaching medical students in hospitals, and researchers have discussed the advantages and disadvantages of the teaching role of junior doctors [35–41]. One study on workplace learning in TBR and LIC models revealed that participating in junior doctor work in a hospital setting may help students transition into their internships more easily in the future [13].. Despite their potential roles in teaching medical students, junior doctors may contribute to the establishment of a hierarchy in a clinical environment. Clinical hierarchies serve as a safety net for patients but as a barrier for medical students [42]. In settings with role-based hierarchies, learning opportunities are allocated on the basis of individuals’ level of training [43]. Therefore, TBR students often have fewer opportunities to participate in meaningful patient care activities because of their peripheral role in a large team. In one study, TBR students described themselves as playing a peripheral and observational role in the clinical environment [13]. The LIC model was designed to overcome the clinical hierarchy and reduce students’ reliance on residents as primary teachers by promoting interactions between students and experienced doctors [13]. In rural placements, LIC students are more likely to be the only trainees and serve as first assistants [44]. In the present study, the students of two LIC programs, in the absence of competing learners (such as interns or residents), not only developed closer relationships with attending physicians but also interacted with patients and preceptors more frequently than the TBR students.

### Strengths and limitations

To the best of our knowledge, our study is the first to compare time spent on clinical activities between two curriculum models and to use ecomaps as primary tools to visualize, quantify, and compare interpersonal relationships and interactions within clinical teams in different curriculum models. Our results may serve as a reference for program directors and researchers in planning future curricula and using ecomaps for social network and interpersonal relationship evaluation.

In addition, our studies provided valuable information about the students’ subjective experience in the new 6-year medical students curriculum in Taiwan. The medical school curriculum in Taiwan was shortened from seven to six years in 2013 [45]. The participants in this study represent the first graduates under this revised curriculum. A previous study had shown no difference in the national OSCE score between the 6- and 7-year curricula [46]; however, the learning experience of students was not well understood. With shortened clinical rotations in the new curriculum, meaningful clinical participation is even more crucial than before. Assigning students from the new education program before confirming their confidence and independent clinical capabilities may lead to exhaustion among medical teams after graduation. We believe that with more understanding of student’s perspectives on workplace engagement and interpersonal interactions, further curriculum optimization could be implemented.

Nevertheless, our study has several limitations. First, the questionnaire used in the present study had not been validated or triangulated, which is similar to other studies examining time spent on clinical activities [47, 48]. However, subjective perceptions of time spent in the workplace may still provide valuable information, as one study reported that considerable amounts of time on activities involving direct patient contact made students feel more positive about their learning environment [48]. Second, because of the cross-sectional design of the study and the use of a self-reported questionnaire, recall bias may occur in the results. Recording a logbook during or after rotation or triangulation by third-party observers may be considered [49]. Third, the small sample size precluded us from conducting correlation analysis or investigating potential causal relationships. For example, we were unable to examine the potential links between interpersonal relationships or the frequency of team member interactions and the time spent on various clinical activities. While the sample size of KH-LIC was relatively small, we conducted additional analyses to compare TS-LIC and TS-TBR only to mitigate the impact of differences between medical institutions (Supplement). These analyses showed similar results to those obtained through three-group comparisons, indicating that the curriculum itself, rather than institution difference, may have been the primary contributing factor. Furthermore, we only collected demographic information regarding gender and age, while the observed differences in other baseline characteristics, such as personality traits, hometown location, or academic performance, may exert a notable influence on the outcomes.

## Conclusion

This study revealed that compared with TBR students, LIC students tend to participate in more patient-oriented activities, such as ward rounds and direct patient care, and have closer relationships and interact more frequently with attending physicians and patients. These findings suggest that the LIC model has unique value and may serve as a reference for program directors looking to improve upon a longstanding model of block rotation-based clerkships.

### Electronic supplementary material

Below is the link to the electronic supplementary material.

Supplementary Material 1

## Data Availability

The datasets generated and analyzed during the current study are not publicly available but are available from the corresponding author on reasonable request.

## Abbreviations

LIC: Longitudinal integrated clerkship
TBR: Traditional block rotation
NDMC: National Defense Medical Center
TS: Tri-Service General Hospita
IM: Internal medicine
KH: Kaohsiung Armed Forces General Hospital
OPD/OR: Outpatient Departments or Operation Room
