# Supplementary material for: Learning through connections: clinical participation and interpersonal relationships in longitudinal integrated clerkships and traditional block rotations in Taiwan
Source: BMC Med Educ. 2024 Feb 10;24:132. doi: 10.1186/s12909-024-05120-y (PMC10859009; doi:10.1186/s12909-024-05120-y)
Supplement: Supplementary file 1 — Supplementary Material 1 [file 12909_2024_5120_MOESM1_ESM.pdf]

Legends

Due to small sample size of KH-LIC, we redo the analysis with TS-LIC and TS-TBR only (Supplement Table 1), which showed similar results to 3 group comparisons. The adjusted analysis for the Relationship an Interaction between two groups (Supplement Table 2) was similar to the result among 3 groups.

Supplement Table1: Comparison between TS-LIC and TS-TBR only

	All			TS-LIC			TS-RBC			MH test				Effect
	Mean	SD	N	Mean	SD	N	Mean	SD	N	MW U	W	Z	P value	size*
**Participation of clinical activities (hrs)**														
Morning meeting	1.02	0.28	44	0.88	0.31	12	1.09	0.27	29	113.5	191.5	-2.07	0.083	0.099
Educational activity	1.47	0.64	43	1.13	0.43	12	1.64	0.68	28	112	190	-1.83	0.102	0.080
Ward round	1.33	0.53	44	1.46	0.62	12	1.28	0.47	29	156	591	-0.58	0.621	0.008
Informal discussion	0.67	0.36	39	0.67	0.25	12	0.64	0.39	24	140	440	-0.17	0.908	0.001
Direct patient care	0.67	0.45	40	1.04	0.54	12	0.46	0.22	26	65.5	416.5	-3.58	0.003	0.328
OPD/OR	1.3	0.99	41	1.8	0.98	12	1.01	0.93	26	78	429	-2.57	0.013	0.166
Senior shadowing	1.19	0.82	44	0.71	0.33	12	1.44	0.89	29	87	165	-2.64	0.012	0.162
Clinical Administration	0.63	0.32	44	0.54	0.26	12	0.68	0.35	29	141	219	-1.19	0.357	0.033
Hand-off	0.44	0.36	36	0.3	0.42	10	0.5	0.34	24	114	169	-0.31	0.838	0.003
Self-directed learning	1.47	0.93	43	1.46	0.96	12	1.54	0.95	28	162.5	240.5	-0.17	0.873	0.001
**Importance (cm, the larger, more important)**														
MS	1.188	.484	41	1.092	.414	12	1.228	.511	29	141.500	219.500	-.941	.347	0.022
I	2.052	.745	29			0	2.052	.745	29					
R	2.703	1.151	33	1.700	.245	4	2.841	1.160	29	16.500	26.500	-2.293	.022	0.164
VS	3.344	1.158	41	2.833	.547	12	3.555	1.281	29	114.000	192.000	-1.723	.085	0.074
NP	2.194	.836	17	2.033	.639	12	2.580	1.188	5	22.000	100.000	-.847	.397	0.045
RN	2.321	1.054	39	1.720	.543	10	2.528	1.114	29	79.000	134.000	-2.128	.033	0.119
**Relationship (cm, the lower, the closer)**														
MS-P	2.411	1.953	41	1.296	.978	12	2.872	2.078	29	97.500	175.500	-2.194	.028	0.120
MS-VS	2.926	2.135	41	.846	.713	12	3.786	1.921	29	25.000	103.000	-4.272	.000	0.456
MS-I	1.400	1.623	29			0	1.400	1.623	29					
MS-R	2.664	2.170	33	2.338	2.180	4	2.709	2.204	29	54.500	64.500	-0.193	.847	0.001
MS-NP	2.275	2.599	16	1.450	1.297	12	4.750	4.088	4	12.000	90.000	-1.462	.144	0.142

MS-RN	3.254	1.884	39	2.600	1.348	10	3.479	2.007	29	108.500	163.500	-1.175	.240	0.036
**Interaction (number of lines, the higher, more frequently)**														
MS-P	2.171	1.424	35	2.833	1.467	12	1.826	1.302	23	73.000	349.000	-2.374	.018	0.166
MS-VS	4.448	3.042	29			0	4.448	3.042	29					
MS-I	2.321	1.278	28	2.667	1.155	3	2.280	1.308	25	28.500	353.500	-0.694	.488	0.018
MS-R	2.278	1.301	36	3.583	1.240	12	1.625	.711	24	22.500	322.500	-4.242	.000	0.514
MS-NP	1.214	.579	14	1.500	.707	2	1.167	.577	12	7.500	85.500	-1.348	.178	0.140
MS-RN	2.846	1.345	13	3.000	1.491	10	2.333	.577	3	11.000	17.000	-0.692	.489	0.040

KH = Kaohsiung Armed Forces General Hospital, TS = Tri-Service General Hospital, LIC = longitudinal integrated clerkship, TBR = traditional block rotation, SD = standard deviation, N = number of students, P = patient, MS = medical student, R = resident, I = intern, RN = registered nurse, NP = nurse practitioner, VS = visiting staff/attending physician. MW U= Mann-Whitney U, W=Wilcoxon W

Supplement Table 2: Comparison between TS-LIC and TS-TBR only, with adjusted method for Relationship and Interaction

	All			TS-LIC			TS-RBC			MH test				Effect size*
	Mean	SD	N	Mean	SD	N	Mean	SD	N	MW U	W	Z	P value	
**Relationship (Z score†)**														
MS-P	.188	.674	41	-.074	.617	12	.297	.677	29	115.500	193.500	-1.676	.094	0.070
MS-VS	.643	1.036	41	-.448	.314	12	1.094	.881	29	24.000	102.000	-4.298	.000	0.462
MS-I	-.406	.733	29			0	-.406	.733	29					
MS-R	.393	.940	33	.967	1.361	4	.314	.870	29	38.000	473.000	-1.103	.270	0.038
MS-NP	.337	.973	16	.153	.922	12	.891	1.036	4	11.000	89.000	-1.576	.115	0.166
MS-RN	.942	.892	39	1.396	.757	10	.785	.893	29	84.000	519.000	-1.962	.050	0.101
**Interaction (Ratio‡)**														
MS-P	.054	.045	35	.088	.052	12	.037	.029	23	27.500	303.500	-3.842	.000	0.434
MS-VS	.096	.050	29			0	.096	.050	29					
MS-I	.051	.029	28	.083	.064	3	.047	.021	25	23.000	348.000	-1.077	.281	0.043
MS-R	.064	.060	36	.123	.072	12	.035	.018	24	10.500	310.500	-4.481	.000	0.574
MS-NP	.072	.029	13	.077	.030	10	.054	.023	3	7.000	13.000	-1.360	.174	0.154
MS-RN	.025	.010	14	.024	.012	2	.025	.011	12	11.000	14.000	-.183	.855	0.003

KH = Kaohsiung Armed Forces General Hospital, TS = Tri-Service General Hospital, LIC = longitudinal integrated clerkship, TBR = traditional block rotation, SD = standard deviation, N = number of students, P = patient, MS = medical student, R = resident, I = intern, RN = registered nurse, NP = nurse practitioner, VS = visiting staff/attending physician.

MW U= Mann-Whitney U, W=Wilcoxon W

† Shortest distances between circles in an ecomap were converted into Z-scores according to the mean and standard deviation of all distances within the ecomap.

‡ Number of the connecting lines between each pair circles in an ecomap was replaced by the ratio of that number to the total number of lines within the ecomap.
